# Role of epithelial splicing regulatory protein 1 in cancer progression

**DOI:** 10.1186/s12935-023-03180-6

**Published:** 2023-12-18

**Authors:** Mi Jeong Kwon

**Affiliations:** 1 Vessel-Organ Interaction Research Center (MRC), College of Pharmacy, Kyungpook National University, Daegu, Republic of Korea; 2 BK21 FOUR KNU Community-Based Intelligent Novel Drug Discovery Education Unit, College of Pharmacy and Research Institute of Pharmaceutical Sciences, Kyungpook National University, 80 Daehak-ro, Buk-gu, Daegu, 41566 Republic of Korea

**Keywords:** Epithelial splicing regulatory protein 1, Alternative splicing, Splicing factor, Epithelial–mesenchymal transition, Dual role, Cancer progression

## Abstract

As aberrant alternative splicing by either dysregulation or mutations of splicing factors contributes to cancer initiation and progression, splicing factors are emerging as potential therapeutic targets for cancer therapy. Therefore, pharmacological modulators targeting splicing factors have been under development. Epithelial splicing regulatory protein 1 (ESRP1) is an epithelial cell-specific splicing factor, whose downregulation is associated with epithelial–mesenchymal transition (EMT) by regulating alternative splicing of multiple genes, such as *CD44*, *CTNND1*, *ENAH*, and *FGFR2*. Consistent with the downregulation of ESRP1 during EMT, it has been initially revealed that high ESRP1 expression is associated with favorable prognosis and ESRP1 plays a tumor-suppressive role in cancer progression. However, ESRP1 has been found to promote cancer progression in some cancers, such as breast and ovarian cancers, indicating that it plays a dual role in cancer progression depending on the type of cancer. Furthermore, recent studies have reported that ESRP1 affects tumor growth by regulating the metabolism of tumor cells or immune cell infiltration in the tumor microenvironment, suggesting the novel roles of ESRP1 in addition to EMT. ESRP1 expression was also associated with response to anticancer drugs. This review describes current understanding of the roles and mechanisms of ESRP1 in cancer progression, and further discusses the emerging novel roles of ESRP1 in cancer and recent attempts to target splicing factors for cancer therapy.

## Introduction

Alternative splicing is a highly regulated process that produces multiple mRNA and protein isoforms from a single gene, and controlled by interaction of RNA-binding proteins (RBPs) with pre-mRNA [1–3]. In human cancers, alternative splicing alterations are frequently observed, and mutations in the splicing regulatory elements of specific cancer-associated genes or changes in the regulatory splicing machinery contribute to cancer initiation and progression by regulating RNA isoforms of genes involved in key cancer-related pathways [4, 5]. A comprehensive analysis of samples from various cancer types revealed that tumor samples have up to 30% more alternative splicing events than normal samples [6]. In this aspect, aberrant alternative splicing regulators are emerging as therapeutic targets of cancer and novel biomarkers that predict response to anticancer agents [4, 7].

Epithelial splicing regulatory protein 1 (ESRP1) is an epithelial cell type-specific splicing factor, whose downregulation is associated with epithelial–mesenchymal transition (EMT) by regulating alternative splicing of several genes such as *CD44*, catenin delta 1 (*CTNND1*) (also known as p120-catenin), *ENAH* (also known as *hMENA*), and fibroblast growth factor receptor 2 (*FGFR2*) [8–11]. While ESRP1 downregulation promotes EMT, ESRP1 overexpression in the mesenchymal state of ovarian or breast cancer cells drives a phenotypic switching from the mesenchymal to epithelial state defined as mesenchymal–epithelial transition (MET), which is an important step for tumor formation in metastatic sites [12–14]. Consistent with the involvement of ESRP1 in the EMT, previous studies have reported the prognostic significance and role of ESRP1 in tumor progression. While ESRP1 has been frequently reported as a tumor suppressor in various cancers [15–18], its pro-tumorigenic role has also been shown in some cancers, such as breast and ovarian cancers [13, 14, 19, 20], indicating that ESRP1 plays a dual role in cancer progression depending on the cancer type. Furthermore, recent studies have demonstrated that ESRP1 is involved in non-EMT processes, thus contributing to cancer progression. ESRP1 has been reported to affect the growth of estrogen receptor (ER)-positive breast cancer by regulating cellular metabolism (fatty acid and lipid metabolism) [21]. ESRP1 expression was also associated with tumor-associated immune cytolytic activity [22] and immunosuppression in the tumor immune microenvironment [14]. A few studies have demonstrated the role of ESRP1 in drug resistance. This review summarizes the current understanding of the roles and mechanisms of ESRP1 in cancer progression, and further discusses the emerging novel roles of ESRP1 in cancer and recent attempts to target splicing factors for cancer therapy.

## Alternative splicing alterations in cancer

Alternative splicing is a critical process of gene regulation that contributes to mRNA complexity and protein diversity in eukaryote [1, 2]. Approximately 90–95% of human multiexon genes undergo alternative splicing [23, 24]. Alternative splicing is a process in which introns are removed from pre-mRNA and exons joined to produce mature mRNA and is catalyzed by the spliceosome, a large RNA–protein complex composed of small nuclear ribonucleoproteins and other numerous proteins [1, 7]. Regulation of alternative splicing is modulated by cis-acting elements within genes known as splicing enhancers and silencers (RNA sequences located in both exon and introns of the pre-mRNA) as well as trans-acting elements mainly consisting of RBPs called splicing factors [3, 25]. Generally, cis-acting elements are classified into exonic splicing enhancers, exonic splicing silencers, intronic splicing enhancers (ISEs), and intronic splicing silencers (ISSs) [26]. Splicing factors bind to cis-acting enhancer or silencer sequences and thus promote exon inclusion or skipping by either enhancing or reducing recruitment of the spliceosome [25]. Serine/arginine-rich (SR) splicing factors and heterogeneous nuclear ribonucleoproteins (hnRNPs) are the two major classes of trans-acting splicing factors; other proteins are also involved in the regulation of alternative splicing [27, 28]. While SR proteins and hnRNPs are ubiquitously expressed across different tissues and cell types, other splicing factors, such as members of the RNA-binding fox-1 homolog (RBFOX), muscleblind-like protein (MBNL), ESRP, and neuro–oncological ventral antigen (NOVA) families, show a cell type-specific pattern of expression [3].

Compared with normal tissues, tumors frequently exhibit alterations in alternative splicing, which produce pro-tumorigenic isoforms of cancer-associated genes related to the hallmarks of cancer such as proliferation, apoptosis, metabolism, invasion, angiogenesis, and immune response, thereby driving cancer progression and metastasis [4, 5, 29]. Recurrent somatic mutations in splicing regulators such as splicing factor 3b subunit 1 (SF3B1), serine/arginine-rich splicing factor 2 (SRSF2), U2 small nuclear RNA auxiliary factor 1, and zinc finger CCCH-type, RNA-binding motif and serine/arginine-rich 2 have been detected in several types of hematological malignancies, whereas few mutations have occurred in solid tumors [4, 5]. In solid tumors, SRs, except SRSF2, hnRNPs, ESRP1/2, and RBFOX2, are overexpressed or downregulated [4, 5]. In particular, the expressions of a number of splicing factors, such as hnRNPs, are frequently upregulated in solid tumors such as breast, colon, and lung cancers, whereas the expressions of RBFOX2 and quaking (QKI) are downregulated [4]. ESRP1/2 have been shown to be down- or upregulated in breast cancer as well as oral/head and neck cancer [4].

## ESRP1 as an EMT regulator

EMT is a process in which epithelial cells acquire mesenchymal properties; this process is important for physiological processes such as development, response to injury, and disease [30, 31]. It enhances cancer cell migration and invasion, thereby promoting cancer progression and metastasis by facilitating the escape of cancer cells from primary tumors [30, 31]. The reverse process, MET, at the sites of metastases is part of metastatic tumor formation [31, 32]. EMT also contributes to the drug resistance and recurrence of cancer by promoting the generation of cancer stem cells or tumor-initiating cells [33, 34]. EMT is a complex process regulated by multiple factors. It is well known to be controlled by transcription factors such as SNAI1/2 (also known as SNAIL/SLUG), twist-related protein 1 (TWIST1) (also known as TWIST), and zinc finger E-box-binding homeobox 1 /2 (ZEB1/2) (also known as δEF1/SIP1), signaling pathways such as transforming growth factor-β (TGF-β), Wnt/β-catenin, and Notch; and microRNA (miRNA) [30]. In addition, accumulating evidence has shown that other mechanisms, such as posttranscriptional RNA processing and posttranslational modifications, are involved in EMT regulation [35]. Alternative splicing is one of the key posttranscriptional RNA processes that regulates EMT [35, 36].

ESRP1 is downregulated during EMT, and ESRP1 depletion in epithelial cells accelerates EMT [8, 9, 37]. Conversely, forced ESRP1 expression in the mesenchymal states of breast [12] or ovarian cancer cells [13, 14] induces a phenotypic switching from the mesenchymal to epithelial state. ESRP1 is involved in EMT or MET by regulating alternative splicing of multiple genes, such as *CD44*, *CTNND1*, *ENAH*, and *FGFR2* [8, 9, 11–13, 38]. Warzecha et al. demonstrated that ESRP1 binds to the GU-rich sequence in ISE/ISS-3, a cis-element regulatory region located in the intron between mutually exclusive exons IIIb and IIIc of *FGFR2* [9]. A comprehensive approach for identifying ESRP-binding motif further demonstrated that ESRP1 binds to a consensus GU-rich motif generally located in the proximal introns of a variable exon [37].

Other splicing factors are also involved in EMT. Similar to ESRP1, ESRP2, RNA-binding motif protein 47 (RBM47) and hnRNPF are epithelial RBPs; they regulate EMT-associated alternative splicing events [9, 39, 40]. Conversely, hnRNPM, RBFOX2, and QKI are examples of mesenchymal RBPs that promote mesenchymal-specific alternative splicing [39, 41–43]. Furthermore, multiple RBPs are known to be involved in a single alternative splicing event [25]. In particular, some splicing factors can antagonize ESRP1-regulated alternative splicing events. While RBM47 generally works to promote epithelial splicing, it can have opposing functions on some ESRP1-regulated alternative splicing targets [39]. RBFOX2 transcriptionally represses ESRP1, and the ratio of ESRP1 and RBFOX2 determines the alternative splicing of *hMENA* in breast cancer [11]. ESRP1 and hnRNPM compete for binding to the same RNA motif and coregulate *CD44* splicing events during EMT [41, 44].

## Dual role of ESRP1 in cancer progression

The prognostic significance and role of ESRP1 in various cancers are described in Table 1 and 2, respectively. In line with the role of ESRP1 in EMT, its high expression is associated with favorable prognosis in some cancers (Table 1). ESRP1 has also been frequently reported to suppress tumor growth or metastasis in various cancers such as lung and pancreatic cancers (Table 2). Patients with high ESRP1 protein expression exhibited a significantly longer overall survival, and ESRP1 attenuated liver metastases in pancreatic cancer in vivo [16]. Furthermore, ESRP1 inhibited the invasion and metastasis of lung adenocarcinoma in vivo [17], and ESRP1 combined with ISG15 synergistically suppressed EMT and lung adenocarcinoma cell invasion [45]. ESRP1 overexpression induced cell apoptosis and cell cycle arrest in small-cell lung cancer cells [46]. In addition, immunohistochemical analysis revealed that the high expression of ESRP1 is significantly associated with favorable prognosis in non-small-cell lung cancer [47]. In colorectal cancer, opposing roles of ESRP1 have been reported. ESRP1 was found to suppress tumorigenic potential in vivo [15], and the levels of ESRP1 mRNA measured using real-time quantitative reverse transcription-polymerase chain reaction correlate with favorable outcomes [48], suggesting that ESRP1 plays a tumor-suppressive role in colorectal cancer. However, another study reported contrasting results that ESRP1 promotes proliferation and tumorigenicity of colorectal cancer cells in vitro and enhances primary tumor growth in vivo [49], and ESRP1 knockdown promotes caspase-independent cell death in colon cancer cells by regulating the translocation of apoptosis-inducing factor [50]. Further studies are warranted to validate the prognostic significance and role of ESRP1 in colorectal cancer. Furthermore, in head and neck squamous cell carcinoma (HNSCC) cells, ESRP1 knockdown enhanced cell motility by affecting the dynamics of the actin cytoskeleton through the induction of Rac1b isoform via regulation of *Rac1* isoform splicing, indicating that ESRP1 suppresses cell migration of HNSCC cells [51]. Analysis of the The Cancer Genome Atlas (TCGA) database revealed a significant association between high *ESRP1* expression and longer patient survival in clear cell renal cell carcinoma [52]. ESRP1 promoted cell cycle G1-phase arrest and inhibited cell proliferation in cervical carcinoma cells by downregulating cyclin A2 protein levels [53]. A recent study reported that alternative splicing of *LRRFIP2* is regulated by ESRP1 and that *LRRFIP2* variant 2, which was dominantly expressed in ESRP1-high cells, decreases the metastatic potential of gastric cancer cells in vitro and in vivo, suggesting the involvement of ESRP1 in the metastatic potential of gastric cancer cells by regulating the isoform switching of *LRRFIP2* [54]. In this study, ESRP1 was found to decrease the migration and invasion of gastric cancer cells, suggesting the tumor-suppressive role of ESRP1. Furthermore, ESRP1 reduction promoted tumor growth and lung metastasis of bladder cancer in vivo [18]. However, the prognostic significance and role of ESRP1 in bladder, cervical, gastric, and renal cancers are unclear and need to be further investigated.

**Table 1:** Prognostic significance of ESRP1 expression in cancer

No	Cancer type	Association of expression with prognosis		Expression level	Method	References
1	Colorectal cancer	Favorable prognosis	High → longer overall survival	Gene	qRT-PCR	[48]
2	Lung cancer (lung adenocarcinoma)	Favorable prognosis	High → absence of metastases, smaller tumor size, and lower clinical stage	Protein	IHC	[17]
3	Lung cancer (non-small-cell lung cancer)	Favorable prognosis	High → longer survival	Protein	IHC	[47]
4	Pancreatic cancer	Favorable prognosis	High → longer overall survival	Protein	IHC	[16]
5	Pancreatic cancer	Favorable prognosis	High → longer survival	Gene	qRT-PCR	[66]
6	Renal cancer (clear cell renal cell carcinoma)	Favorable prognosis	High → longer survival	Gene	Public database analysis (TCGA)	[52]
7	Breast cancer	Poor prognosis	High → shorter overall survival	Gene	Public database analysis (GEO dataset)	[19]
8	Breast cancer	Favorable prognosis	High → longer survival	Gene	Public database analysis (TCGA)	[52]
9	Breast cancer	Poor prognosis	High → shorter distant metastasis-free survival	Gene	Public database analysis (Kaplan − Meier plotter)	[20]
10	Breast cancer (ER-positive breast cancer)	Poor prognosis	High → shorter overall survival	Gene	Public database analysis (BreastMark + TCGA)	[21]
11	Breast cancer	Poor prognosis	High → shorter relapse-free survival	Gene	Public database analysis (Kaplan − Meier plotter)	[55]
12	Ovarian cancer	Poor prognosis	High → progression-free survival	Gene	Public database analysis (TCGA)	[13]
13	Prostate cancer	Poor prognosis	High → shorter recurrence-free survival	Gene	Public database analysis (KM-express)	[61]
14	Prostate cancer	Poor prognosis	High → shorter biochemical recurrence-free survival, shorter cancer-specific survival	Protein	IHC	[58]
15	Prostate cancer	Poor prognosis	High → shorter PSA recurrence-free survival	Protein	IHC	[59]
16	Prostate cancer	Poor prognosis	High → biochemical recurrence-free survival	Protein	IHC	[60]
17	Skin cancer (melanoma)	Poor prognosis	ESRP-low tumors → trend of more favorable survival	Gene	Public database analysis (TCGA)	[22]
18	Skin cancer (cutaneous malignant melanoma)	Poor prognosis	High → shorter overall survival	Gene	Public database analysis	[62]

*ER* estrogen receptor; *GEO*, Gene Expression Omnibus, *IHC*, immunohistochemistry, *qRT-PCR* real-time quantitative reverse transcription-polymerase chain reaction, *TCGA* The Cancer Genome Atlas

**Table 2:** Role of ESRP1 in cancer progression

No	Cancer type	Roles	Functions	References
1	Bladder cancer	Tumor suppressive	ESRP1 decreases cell growth and migration (in vitro) ESRP1 inhibits xenograft tumor formation and reduces the occurrence of lung metastasis (in vivo)	[18]
2	Cervical cancer	Tumor suppressive	ESRP1 induces cell cycle G1-phase arrest and inhibits cell proliferation (in vitro)	[53]
3	Colorectal cancer	Tumor suppressive	ESRP1 inhibits anchorage-independent growth (in vitro) ESRP1 suppresses tumorigenic potential (in vivo)	[15]
4	Colorectal cancer	Tumor promoting	ESRP1 promotes proliferation and tumorigenicity (in vitro) ESRP1 enhances primary tumor growth (in vivo)	[49]
5	Colorectal cancer	Tumor promoting	ESRP1 knockdown promotes caspase-independent cell death (in vitro)	[50]
6	Gastric cancer	Tumor suppressive	ESRP1 decreases migration and invasion (in vitro)	[54]
7	Head and neck cancer	Tumor suppressive	ESRP1 decreases cell motility (in vitro)	[51]
8	Lung cancer (lung adenocarcinoma)	Tumor suppressive	ESRP1 knockdown enhances invasion (in vitro) ESRP1 knockdown suppresses tumor growth and liver metastasis (in vivo)	[17]
9	Lung cancer (small-cell lung cancer)	Tumor suppressive	ESRP1 induces cell apoptosis and cell cycle arrest (in vitro) ESRP1 inhibits tumor growth (in vivo)	[46]
10	Pancreatic cancer	Tumor suppressive	ESRP1 decreases migration and invasion (in vitro) ESRP1 attenuates liver metastases (in vivo)	[16]
11	Prostate cancer	Tumor suppressive	ESRP1 inhibits tumor growth (in vivo)	[61]
12	Breast cancer	Tumor promoting	ESRP1 promotes lung metastasis (in vivo)	[19]
13	Breast cancer	Tumor promoting	ESRP1 promotes lung metastasis (in vivo)	[20]
14	Breast cancer (ER-positive breast cancer)	Tumor promoting	ESRP1 knockdown inhibits cell growth (in vitro and in vivo)	[21]
15	Breast cancer	Tumor suppressive	ESRP1 knockdown increases invasion (in vitro)	[11]
16	Breast cancer	Tumor suppressive	ESRP1 decreases tumor cell contractility and intravasation (in vitro and in vivo)	[56]
17	Ovarian cancer	Tumor promoting	ESRP1 increases cell proliferation and decreases migration (in vitro)	[13]
18	Ovarian cancer	Tumor promoting	ESRP1 inhibits cell migration and invasion but promotes colonization (in vitro) ESRP1 enhances tumor growth (in vivo)	[14]

*ER* estrogen receptor

Contrarily, the negative association between *ESRP1* expression and patient survival as well as the pro-tumorigenic role of ESRP1 have been reported in some cancers, such as breast and ovarian cancers (Tables 1 and 2). In breast cancer, analysis of the Gene Expression Omnibus database revealed that high *ESRP1* expression is correlated with significantly shorter overall survival in patients with breast cancer [19]. Other studies using the Kaplan–Meier plotter have shown that high *ESRP1* expression is associated with shorter distant metastasis-free survival or relapse-free survival [20, 55]. In particular, in public database analysis using BreastMark and TCGA data, *ESRP1* expression was significantly associated with shorter overall survival in ER-positive, but not ER-negative, breast cancer [21], indicating the subtype-specific prognostic significance of *ESRP1* expression. Consistent with the prognostic significance of *ESRP1* in breast cancer, ESRP1 was demonstrated to promote lung metastasis in orthotopic mouse model of breast cancer by regulating alternative splicing of *CD44* mRNA [19, 20]. However, Lu et al. reported a significant association between high *ESRP1* expression and longer survival of patients with breast cancer [52]. ESRP1 downregulation enhanced invasion of breast cancer cells by promoting the generation of *hMENAΔ11a* isoform, which results in the mesenchymal phenotype [11]. Furthermore, a recent study by Wang et al. demonstrated that stiff matrix promotes the intravasation of breast cancer cells through the decreased ESRP1-mediated alternative splicing of *MENA*, indicating that ESRP1 decreases breast cancer cell intravasation [56]. These contrasting results for ESRP1 in breast cancer may be partly attributed to the different subtypes or stages of breast cancer used in the studies. Further studies are warranted to validate the prognostic value and role of ESRP1 in subtypes of breast cancer. ESRP1 has been shown to play a pro-tumorigenic role in ovarian cancer. Analysis of the TCGA database revealed that high *ESRP1* expression is significantly associated with shorter progression-free survival and that ESRP1 overexpression drives MET in association with the upregulation of *CDH1* expression and alternative splicing of *CD44* and *ENAH*, but not *FGFR2*, in ovarian cancer cells [13]. Deng et al. also reported that ESRP1 induces MET and promotes colonization in ovarian cancer cells [14]. They further demonstrated that ESRP1 overexpression promotes tumor growth and metastasis in vivo, which is associated with the immunosuppressive effect of ESRP1 in the tumor immune microenvironment.

In prostate cancer, integrative genomic analyses of prostate tumors identified that amplification of *ESRP1* is associated with early-onset aggressive prostate cancer and high *ESRP1* expression correlates with a more proliferative gene expression profile [57]. In addition, several immunohistochemical analyses demonstrated that ESRP1 expression is significantly associated with poor prognosis in prostate cancer [58–60], suggesting that ESRP1 plays a pro-tumorigenic role. However, contrary to the negative prognostic significance of ESRP1, ESRP1 has been shown to inhibit the growth of prostate cancer xenografts using androgen receptor-negative prostate cancer cells in vivo [61]. Public database analyses revealed the association between high *ESRP1* expression and poor prognosis of melanoma [22, 62]; however, the role of ESRP1 in melanoma remains unknown. Further investigation on the role of ESRP1 in prostate cancer and melanoma are also warranted.

## Functional differences between ESRP1 and ESRP2

Similar to ESRP1, ESRP2 is associated with epithelial phenotype and its downregulation promotes EMT by regulating alternative splicing of multiple genes [9]. While ESRP1 and ESRP2 share similar structure features and play similar roles in cancers, they may function differently [63, 64]. Ishii et al., revealed that both ESRP1 and ESRP2 knockdown increases the motility of HNSCC cells, but they act through distinct mechanisms [51]. Knockdown of ESRP1 modulated the dynamics of the actin cytoskeleton through the regulation of *Rac1* isoform splicing, whereas knockdown of ESRP2 decreased cell–cell adhesion by upregulating EMT-related transcription factors δEF1 and SIP1. This study also showed that their effect on the alternative splicing of target gene is different. ESRP1 knockdown, but not ESRP2, induced a switching from the *CD44 variant (CD44v)* isoform to the *CD44 standard (CD44s)* isoform in HNSCC cells [51]. In another study, in normal mouse mammary gland epithelial cells with low ESRP1 expression, ESRP2 knockdown affected an isoform switching of *CD44* [65]. These results suggest that the effect of ESRP1 or ESRP2 on target pre-mRNAs is different depending on their endogenous expression levels, cell type and target pre-mRNAs. However, it is not known whether ESRP1 and ESRP2 interact to play a role in cancer progression, and further studies are warranted to elucidate the interplay between ESRP1 and ESRP2.

## Role of ESRP1 in response to anticancer drugs

Given that EMT is involved in drug resistance by generating cancer stem cells or tumor-initiating cells [33, 34], ESRP1 is expected to play a role in response to anticancer drugs and stemness. Consistent with the role of ESRP1 in EMT and stemness, ESRP1 overexpression increased the sensitivity of small-cell lung cancer cells to chemotherapy by regulating alternative splicing of *CARM1*, thereby inhibiting TGF-β/Smad signaling [46]. Similarly, the expression of ESRP1 enhanced the sensitivity of pancreatic cancer cells to gemcitabine [66]. Furthermore, a recent study revealed that ESRP1 overexpression in paclitaxel-resistant population of triple-negative breast cancer cells increases their sensitivity to paclitaxel by regulating *α6 integrin* splicing [67]. Notably, ESRP1 regulated breast cancer stem cell properties by determining the expression of stemness-inducing *α6B* variant relative to *α6A* variant [68]. ESRP1 was also found to suppress breast cancer stem cell function by promoting *CD44* splice isoform switching from *CD44s* isoform that causes stemness to *CD44v* isoform [69]. Importantly, the expression of *ESRP1* was associated with response to immunotherapy in cancer. In melanoma, low *ESRP1* expression was correlated with greater tumor-associated immune cytolytic activity and patients with low *ESRP1* expression showed a favorable survival, suggesting the potential utility of *ESRP1* as a biomarker in predicting response to immunotherapy [22]. Patients treated with tamoxifen with high *ESRP1* expression had a significantly shorter overall survival (using the BreastMark microarray platform), and ESRP1 knockdown significantly inhibited tumor growth in vivo in endocrine-resistant ER-positive breast cancer [21]. This suggests the association of ESRP1 with endocrine resistance. However, it is unclear whether ESRP1 promotes endocrine resistance in ER-positive breast cancer. Thus, further studies are warranted to elucidate the role of ESRP1 in endocrine resistance.

## Emerging roles of ESRP1 in cancer

ESRP1 itself was reported to be alternatively spliced to produce isoforms with distinct nuclear or cytoplasmic localization [70]. While the role of nuclear ESRP1 in the regulation of alternative splicing has been extensively studied, the role of ESRP1 in the cytoplasm has been less well understood. A few studies have shown that ESRP1 in the cytoplasm is involved in non-EMT process by controlling mRNA translation. Esrp1 knockdown in the mouse embryonic stem cells enhanced the self-renewal of these cells by increasing the expression of pluripotency-related factors such as Oct4 and Sox2 through the direct binding to the 5’ untranslated region (UTR) of pluripotency-related mRNAs [71], suggesting that ESRP1 acts as a regulator of self-renewal. Furthermore, a recent study demonstrated that a loss of cytoplasmic ESRP1 causes an increase in the protein expression of CTNND1 by binding the 3’ UTR of CTNND1 without affecting the mRNA level [72]. This suggests a role of cytoplasmic ESRP1 in the epithelial cell function through posttranscriptional regulation.

A novel role of ESRP1 in cancer progression through posttranscriptional regulation of cancer-associated genes has also been reported. Leontieva et al. revealed that ESRP1 functions as a tumor suppressor in colon cancer cells by regulating the mRNA translation of cancer-related genes such as c-Myc and Fos through the binding to their 5’ UTR [15]. ESRP1 was shown to induce G1-phase cell cycle arrest and suppress cell proliferation in cervical cancer cells by decreasing cyclin A2 mRNA stability through the direct binding to the 3’ UTR of cyclin A2 [53]. Gokmen-Polar et al. demonstrated that ESRP1 knockdown inhibits endocrine-resistant ER-positive breast cancer growth by regulating cellular metabolism without developing a mesenchymal phenotype [21]. In particular, in the study by Gokmen-Polar et al., ESRP1 knockdown decreased the expression of enzymes related to lipid metabolism and oxidoreductase processes, including fatty acid synthase, stearoyl-CoA desaturase 1, and phosphoglycerate dehydrogenase (PHGDH), at both the mRNA and protein levels and increased basal respiration and spare respiration capacity. This suggests that ESRP1 affects tumor progression through the dysregulation of cellular metabolism. Furthermore, ESRP1 was found to regulate PHGDH expression by binding to the 5′ UTR of PHGDH, thereby increasing its mRNA stability in hormone therapy-resistant ER-positive breast cancer [73], supporting the novel mechanism of ESRP1.

**Fig. 1:**
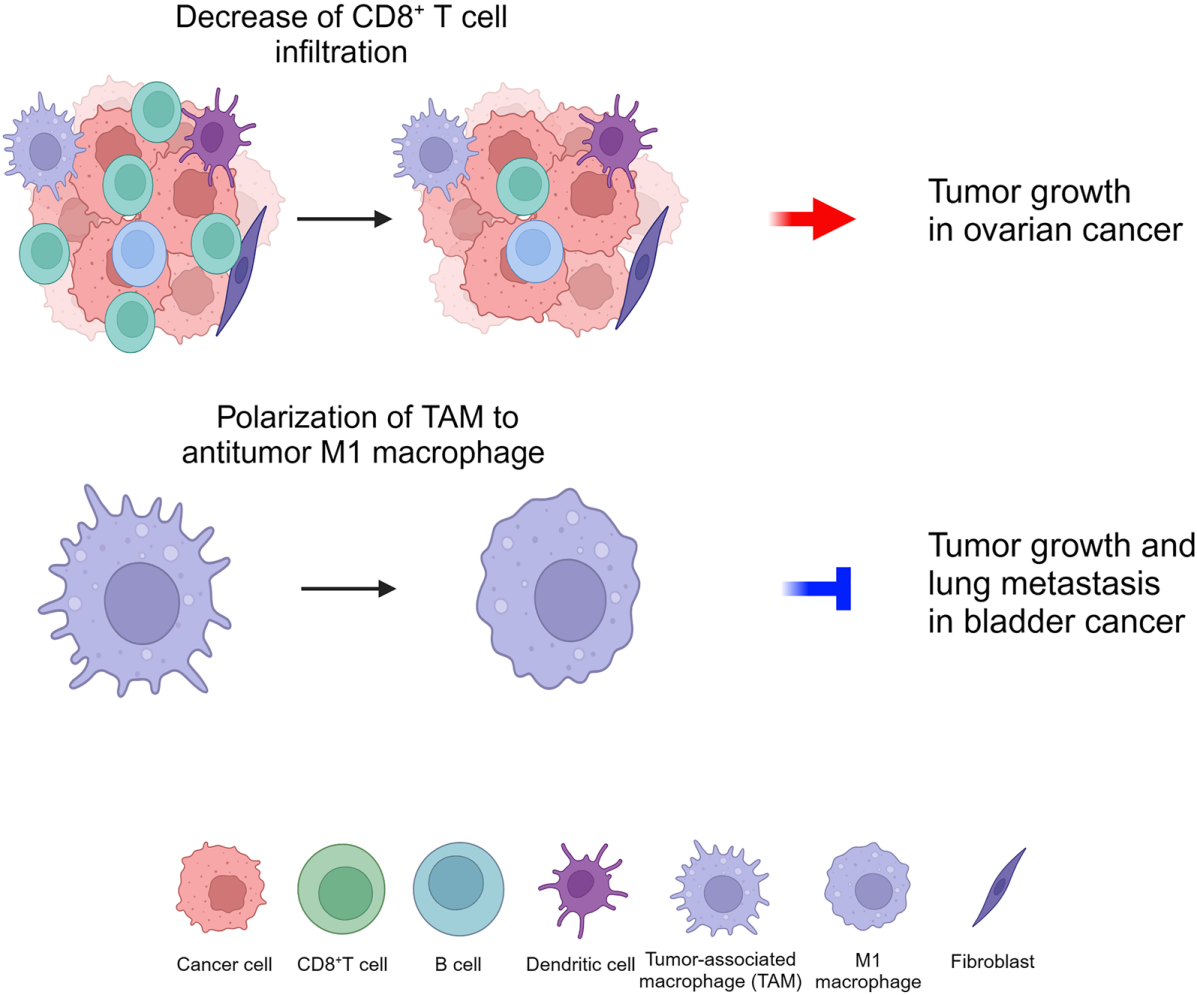
Role of ESRP1 in immune regulation in the tumor microenvironment. ESRP1 plays a pro-tumorigenic role in ovarian cancer by decreasing infiltration of CD8^+^ T cells in the tumor microenvironment. Conversely, ESRP1 suppresses tumor growth and lung metastasis in bladder cancer in association with polarization of tumor-associated macrophage into antitumor phenotype

Importantly, ESRP1 was involved in the regulation of immune cells in the tumor microenvironment (TME) (Fig. 1). To determine the effect of ESRP1 in the tumor immune microenvironment in vivo in ovarian cancer, ESRP1-overexpressing ovarian cancer cells or empty vector-transfected cells were subcutaneously injected into mice, and infiltrating CD8^+^ T cells in tumor nodules were observed [14]. This study found that the ESRP1-overexpressing group has significantly heavier tumor weight and lower infiltrated CD8^+^ T cells than the empty vector group [14]. These results indicate that ESRP1 decreases CD8^+^ T cell infiltration in the TME, thereby promoting tumor growth. Another study reported the effect of ESRP1 on tumor-associated macrophages in the TME. ESRP1 inhibited tumor growth and lung metastasis in xenograft mouse model of bladder cancer, and also promoted the polarization of tumor-associated macrophages into anti-tumor phenotype in vivo [18]. This suggests that ESRP1 plays a tumor-suppressive role in bladder cancer in part by its effect on macrophage polarization.

## ESRP1 regulation in cancer

**Fig. 2:**
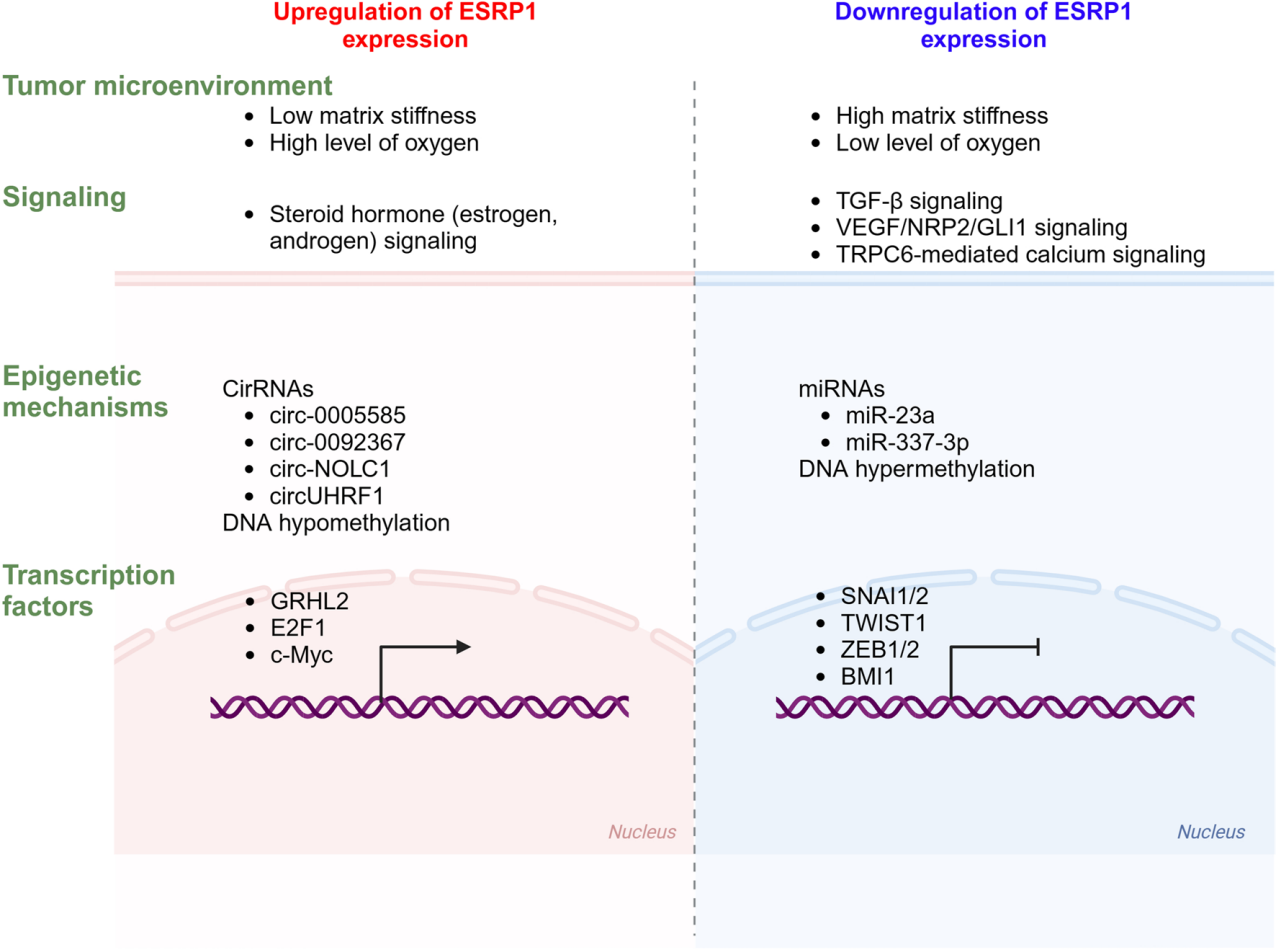
Regulation of ESRP1 expression. ESRP1 expression is regulated by multiple factors such as transcription factors, epigenetic mechanisms, signaling and tumor microenvironment including oxygen and matrix stiffness at transcriptional and posttranscriptional levels cirRNA, circular RNA; GLI1, GLI family zinc finger 1; GRHL2, grainyhead like transcription factor 2; miRNA, microRNA; NRP2, neuropilin-2; TGF-β, transforming growth factor-β; TRPC6, transient receptor potential channel 6; TWIST1, twist-related protein 1; VEGF, vascular endothelial growth factor; ZEB1/2, zinc finger E-box-binding homeobox 1

Based on ESRP1 downregulation during EMT, several studies have been conducted to elucidate the mechanisms downregulating ESRP1 expression. EMT-related transcription factors have been shown to repress the transcriptional levels of ESRP1 (Fig. 2). EMT induction by twist led to the downregulation of *ESRP1* mRNA [9]. Snail and ZEB1 directly bind to the promoter region of *ESRP1*, resulting in the transcriptional repression of ESRP1 during EMT [10, 74]. Double knockdown of δEF1 and SIP1 increased the mRNA levels of ESRP1 in human breast cancer cells [65]. SLUG knockdown caused an increase in *ESRP1* expression, and TGF-β-induced SLUG upregulation led to the transcriptional downregulation of ESRP1 in breast cancer cells, indicating that SLUG acts as a transcriptional repressor of ESRP1 [11]. Treatment with TGF-β repressed the ESRP1 at the mRNA and protein levels in cells derived from mammary gland epithelial cells [65]. Vascular endothelial growth factor (VEGF)/neuropilin-2 (NRP2)/GLI family zinc finger 1 (GLI1) signaling suppressed ESRP1 expression via transcription repressor polycomb complex protein BMI1 in triple-negative breast cancer cell lines [68]. A recent study showed that transient receptor potential channel 6 (TRPC6)-mediated calcium entry induces the expression of *integrin α6B* splice variant by repressing ESRP1, which regulates response to chemotherapy in triple-negative breast cancer cells, indicating the downregulation of ESRP1 by TRPC6-mediated calcium signaling [67]. Epigenetic mechanisms, including noncoding RNAs, have also been found to regulate ESRP1 expression (Fig. 2). miR-23a increased alternative splicing of *CD44*v to *CD44*s and *FGFR2* IIIb to IIIc via downregulation of ESRP1 expression by binding to its 3’ UTR, thereby promoting EMT and metastasis in pancreatic cancer [75]. miR-337-3p inhibited breast cancer cell migration and invasion by downregulating ESRP1 [76]. Notably, TME also affects ESRP1 expression, which is oxygen-dependent. Hypoxia-induced TGF-β signaling upregulates SLUG and RBFOX2, which in turn transcriptionally repress ESRP1 expression; furthermore, ESRP1 downregulation promotes the generation of the *hMENAΔ11a* isoform, which results in the mesenchymal phenotype and thereby promotes breast cancer cell invasion [11]. In addition, Wang et al., revealed that stiff matrix promotes tumor cell intravasation by regulating *MENA* splicing via ESRP1 downregulation in breast cancer [56]. This study also demonstrated that increased matrix stiffness decreases ESRP1 expression, whereas focal adhesion kinase (FAK) inhibition increases ESRP1 expression, suggesting that ESRP1 expression regulated by matrix stiffness is mediated by FAK-mediated mechanotransduction. These results indicate that hypoxic and stiff matrix TME negatively downregulate ESRP1 expression (Fig. 2).

Contrarily, the positive regulation mechanism of ESRP1 expression is poorly understood, whereas the transcription factor grainyhead like transcription factor 2 (Grhl2) was shown to increase *Esrp1* expression in a mouse mammary carcinoma cell line [77] and DNA hypomethylation of CpG sites in the *ESRP1* promoter region was found to be significantly correlated with high *ESRP1* expression in ovarian cancer cells [13] (Fig. 2). However, recent studies have demonstrated the mechanisms upregulating ESRP1 expression in cancer cells. Several circular RNAs (circRNAs) increased ESRP1 expression (Fig. 2). circ-0005585 upregulated ESRP1 expression by sponging miR-23a/b and miR-15a/15b/16 in ovarian cancer cells [14]. Similarly, circ-0092367 overexpression increased the protein levels of ESRP1 in pancreatic cancer cells by serving as a miR-1206 sponge [66]. circ-NOLC1 bound to ESRP1, and circ-NOLC1 overexpression significantly increased the levels of ESRP1 protein and mRNA in ovarian cancer cells [78]. Furthermore, the transcription factor c-Myc which is positively regulated by circUHRF1 promoted the transcription levels of ESRP1 in oral squamous cell carcinoma cells, indicating that circUHRF1 is involved in the upregulation of ESRP1 [79]. *ESRP1* expression was increased in colorectal cancer cells expressing wild-type full-length adenomatous polyposis coli (APC) and was correlated with APC levels in colorectal cancer primary tumors [80]. In this study, treatment with a Wnt signaling inhibitor increased *ESRP1* expression, suggesting that intact APC inhibits Wnt/β-catenin signaling and thereby upregulates *ESRP1* expression. Furthermore, Ashok et al. investigated the mechanism that upregulates ESRP1 expression during breast carcinogenesis and found that elevated levels of the transcriptional activator E2F1 and increased CpG hydroxymethylation at the E2F1 binding site in the *ESRP1* promoter enhances ESRP1 expression in breast cancer [81]. However, the study by Ashok et al. also demonstrated that under hypoxia, ESRP1 expression is downregulated by decreased DNA hydroxymethylation and increased DNA methylation levels at the E2F1 binding site in the *ESRP1* promoter. This was found to be due to hypoxia-driven reduced activity of tet methylcytosine dioxygenase 3 and increased activity of de novo DNA methyltransferases (DNMT3A and 3B), suggesting that oxygen-dependent epigenetic modifications is crucial in the regulation of ESRP1 expression in breast cancer. Taken together, these results indicate that multiple factors, including TME, epigenetic mechanisms, and transcription factors, together play a role in ESRP1 regulation during cancer progression. In hormone-dependent cancers such as breast and prostate cancers, *ESRP1* expression was shown to be associated with steroid hormone signaling. Public transcriptome and chromatin immunoprecipitation-sequencing data analyses revealed that *ESRP1* expression correlates with *ESR1* expression in breast cancer, and ERα binding sites are located within the *ESRP1* promoter, suggesting that *ESRP1* expression is regulated by ERα signaling in breast cancer [82]. Similarly, androgen levels were found to regulate *ESRP1* expression. RNA-sequencing data from human prostate cancer showed that *ESRP1* expression is significantly downregulated following androgen deprivation therapy [61].

## Targeting splicing factors in cancer

As alternative splicing alterations by mutations or dysregulations in splicing factors are involved in cancer initiation and progression, splicing factors have been considered as potential therapeutic targets for cancer therapy. In particular, several splicing factors, such as hnRNP A2/B1, SRSF1, and SRSF6, have been shown to act as driver oncogenes in some cancers [83–86]; thus, they have gained attention as promising targets for cancer therapy. Strategies targeting core spliceosome assembly inhibit the early stages of spliceosome assembly and are likely to cause nonspecific and toxic effects, and focusing on the direct inhibition of specific splicing factors is expected to exert a more specific effect with less toxicity than targeting core spliceosome assembly [28].

Recently, several therapeutic strategies targeting splicing factors, including small molecule and oligonucleotide-based molecules, have been identified. The possible strategies that use small molecules include 1) inhibition of kinase that phosphorylates a splicing factor, 2) inhibition of a splicing factor by ubiquitination and degradation, 3) direct inhibition of a splicing factor, 4) inhibition of a splicing factor by targeting specific RNA recognition motif to interfere with its RNA-binding activity, and 5) direct binding of small molecule to a splicing factor for allosteric modulation to either inhibit or activate the splicing factor [28]. SR proteins are phosphorylated by several kinases, including two major regulators, SR protein kinase (SRPK) [87, 88] and CDC2-like kinase (CLK) [89]. Extensive screening for chemical compounds that inhibit SR protein phosphorylation led to the identification of CLK inhibitors and/or SRPK inhibitors. TG003, a CLK inhibitor that exerts a potent inhibitory effect on Clk1, inhibited SF2/ASF-dependent splicing of β-globin pre-mRNA in vitro by suppressing Clk1-mediated phosphorylation [90]. Furthermore, inhibition of CLK activity was associated with activity for regulating RPS6KB1 (S6K) splicing and inhibiting cancer cell growth, suggesting that CLK inhibitors suppress cancer cell growth by altering S6K pre-mRNA splicing [91]. One example of the currently suggested strategy to use oligonucleotide-based molecules is to design decoy oligonucleotides, which directly bind to splicing factors. Decoy oligonucleotides inhibit only the splicing factor activity without interfering with other activities by binding to the RNA-binding domain of the splicing factor [92]. Denichenko et al. designed decoy oligonucleotides for three splicing factors, RBFOX1/2, polypyrimidine tract binding protein 1 (PTBP1), and SRSF1 [92]; their expressions are altered in various cancer types and are known to be involved in cancer progression [4, 83, 85, 93, 94]. They demonstrated that inhibition of PTBP1 with decoy oligonucleotide can suppress the oncogenic properties of breast and glioblastoma cancer cells [92].

However, the oncogenic role of ESRP1 in cancer remains unclear, whereas some studies have reported its pro-tumorigenic roles in some cancers, including breast and ovarian cancers. Further efforts to increase understanding on the roles and mechanism of ESRP1 in tumorigenesis are necessary for targeting ESRP1 for cancer therapy.

## Conclusions

As aberrant alternative splicing by either dysregulation or mutations of splicing factors has been shown to be involved in cancer development and progression, splicing factors are emerging as potential therapeutic targets for cancer therapy. Therefore, recent attempts have been made to develop pharmacological modulators of splicing factors. ESRP1 is a key epithelial cell-specific splicing factor that acts as a master regulator of EMT. ESRP1 has been initially considered as a tumor suppressor. However, the pro-tumorigenic roles of ESRP1 have been revealed in some cancers, such as breast and ovarian cancers. Furthermore, recent studies have demonstrated that ESRP1 is involved in cancer progression by regulating cellular metabolism or immune cell infiltration in the TME, indicating the novel roles of ESRP1 in addition to EMT. Despite the prognostic significance and role in some cancers, the role and underlying mechanism of ESRP1 in cancer progression remain unclear. Additional studies are warranted to validate whether ESRP1 is suitable as a therapeutic target for cancer therapy or potential biomarker for prognosis or predicting response to anticancer drugs. However, given that ESRP1 plays a dual role in cancer progression, ESRP1 itself may not be a suitable therapeutic target in some cancers, and it is required to consider other effective approaches including targeting its downstream proteins.

## Data Availability

Not applicable.

## Abbreviations

RBP: RNA-binding protein
ESRP1: Epithelial splicing regulatory protein 1
EMT: Epithelial–mesenchymal transition
CTNND1: Catenin delta 1
FGFR2: Fibroblast growth factor receptor 2
MET: Mesenchymal–epithelial transition
ER: Estrogen receptor
ISE: Intronic splicing enhancer
ISS: Intronic splicing silencer
SR: Serine/arginine-rich
hnRNP: Heterogeneous nuclear ribonucleoprotein
RBFOX: Rna-binding fox-1 homolog
MBNL: Muscleblind-like protein
NOVA: Neuro–oncological ventral antigen
SF3B1: Splicing factor 3b subunit 1
SRSF2: Serine/arginine-rich splicing factor 2
QKI: Quaking
TWIST1: Twist-related protein 1
ZEB1/2: Zinc finger E-box-binding homeobox 1/2
TGF-β: Transforming growth factor-β
miRNA: MicroRNA
RBM47: RNA-binding motif protein 47
HNSCC: Head and neck squamous cell carcinoma
TCGA: The Cancer Genome Atlas
CD44v: CD44 variant
CD44s: CD44 standard
UTR: Untranslated region
PHGDH: Phosphoglycerate dehydrogenase
TME: Tumor microenvironment
VEGF: Vascular endothelial growth factor
NRP2: Neuropilin-2
GLI1: Gli family zinc finger 1
TRPC6: Transient receptor potential channel 6
FAK: Focal adhesion kinase
Grhl2: Grainyhead like transcription factor 2
circRNA: Circular RNA
APC: Adenomatous polyposis coli
SRPK: SR protein kinase
CLK: CDC2-like kinase
PTBP1: Polypyrimidine tract binding protein 1
